# Computational and experimental approaches to the molecular structure of the HCl adduct of Me_3_PO

**DOI:** 10.1016/j.crci.2010.07.006

**Published:** 2010-08

**Authors:** Andreas Orthaber, Ferdinand Belaj, Rudolf Pietschnig

**Affiliations:** Department of Chemistry, Inorganic Section, University of Graz, Schubertstr. 1, 8010 Graz, Austria

**Keywords:** Phosphorus, Donor–acceptor systems, Hydrogen bonds, Ion pairs, Solvent effects, *Ab initio* calculations, X-ray diffraction, Phosphore, Systèmes donneur–accepteur, Liaisons hydrogènes, Paires ioniques, Effets de solvant, Approche DFT, Diffraction X

## Abstract

The reaction of anhydrous HCl_(g)_ with trimethyl phosphane oxide yields trimethylhydroxy phosphonium chloride. A crystal structure analysis showed that the prevalent mesomeric structure in the solid state is the phosphonium chloride ion pair. *Ab initio* calculations in the gas phase cannot reproduce these findings, whereas higher correlated methods (CISD) and solvation models predict the experimental structure correctly.

## Résumé

La réaction du HCl_(g)_ anhydre avec l’oxyde de triméthylphosphine conduit au chlorure d’hydroxytriméthylphosphonium. L’analyse par diffraction des rayons X indique que la forme prédominante est une paire ionique. Les approches DFT en phase gazeuse ne permettent pas de retrouver la structure déterminée à l’état cristallin, alors que les méthodes plus hautement corrélées CISD ainsi que des modèles de solvatation reproduisent la structure expérimentale.

## 1 Introduction

**Scheme 1:**
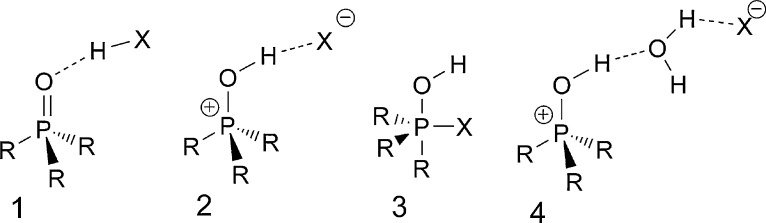
Isomeric structures of hydrogen halide adducts of oxophosphoranes **1**–**3**, including a water containing variety (**4**).

The bonding situation in different phosphanes, phosphane oxides, and ylides has been investigated over the last decades [1–3]. Earlier investigations of differently substituted formal “hydroxy phosphonium halides” showed the influence of the counter ion with respect to the contributions of the mesomeric structures 1,2 and their isomer 3 (Scheme 1). The corresponding triphenyl substituted phosphonium cation was studied crystallographically with different formal counter ions, like for e.g. F^−^, [4], Cl^−^ [5], Br^−^ [6], H_2_O*Br^−^ [7]. For X = F the best description is formula **1**, which can be attributed to the strong H–F bond. For the softer atoms (X = Cl and Br), which are also weaker bases, the crystal structures revealed the corresponding hydroxytriphenyl phosphonium halides **(2)** as the prevalent tautomeric structure. For X = Br also, the formation of a water adduct (type **4**) was observed. Whereas the phenyl substituted phosphonium compounds have been studied extensively, only little is known about their aliphatic congeners. Some earlier spectroscopic results from NMR and IR investigations suggested, however, a similar trend for the aliphatic compounds [8]. Nevertheless, only a single crystal structure with trialkyl substitution was reported in the literature, so far (type **2**: R = ^*i*^Pr, X = I) [9], and a further compound having an indenyl and two methyl substituents in the form of its water adduct (type **4**: X = Cl) [10]. By contrast, the *soft–soft* interactions of various iodo-phosphonium salts with iodine (I^−^, I_3_^−^) have been studied extensively [9,11–13]. In our work, we wanted to investigate the bonding situation of adducts of hydrogen halides with the smallest trialkyloxophosphorane, *i.e.* trimethyl phosphane oxide.

## 2 Results and discussion

In order to get insight into the bonding situation of the alkyl substituted compounds R_3_POHX, we performed an exploratory *ab initio* study on the simplest representatives (R=CH_3_, X = F, Cl, Br). Our initial attempts to analyze these molecules by means of DFT calculations (B3LYP) with a triple ζ basis set (6–311G**) revealed that only the bromine derivative exists as a type **2** structure. We attributed these findings to the incompleteness of the basis set not able to describe diffuse electron distribution. Addition of diffuse functions (B3LYP/6-311++G**) did not alter the findings. The fluorine and the bromine derivatives show the phosphane oxide adduct (**2**) and the phosphonium ion (**1**), respectively. A summary of selected geometric parameters is given in Table 1. Nevertheless, the PO bond lengths for the fluorine derivative (1.513 Å) is significantly longer than for trimethylphosphane oxide (1.489(6) Å [14] and 1.500 Å) suggesting some interaction between the P=O and the HF moiety.^1^ Interestingly, for the chlorine derivative, the calculations also predict the HCl adduct rather than the phosphonium ion as the minimum energy structure. Interaction of the HCl molecule with trimethylphosphane oxide results in a stabilization by ca. 3.2 kcal/mol (Δ*G*_interaction_). Only the bromine derivative shows the expected phosphonium ion structure having a shortened O–H (1.069 Å) and elongated H–Br (1.935 Å) distance.

**Scheme 2:**
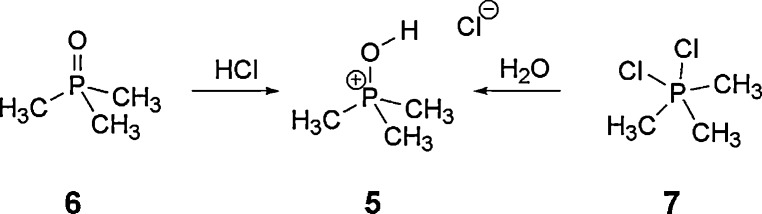
Synthesis of **5** starting from trimethyl phosphane oxide (**6**) or the chlorotrimethyl phosphonium chloride (**7**).

This is, in fact, the opposite of the experimental findings with R = phenyl. It seems, however, very unlikely that changing phenyl to alkyl substituents would switch the preferred structure from type **2** to **1**. Therefore, we synthesized this compound to check the bonding situation experimentally. General access to phosphonium halides is given by reaction of the phosphane oxide **6** with the corresponding anhydrous HX. We were also able to obtain **5** after gentle hydrolysis of **7** [15] (Scheme 2). In the ^31^P NMR spectra, a signal at +66.9 ppm is observed. This is in good agreement with similar compounds [16]. The proton spectra show one doublet at 1.93 ppm (^3^*J*_PH_ = 18 Hz) and a broad singlet at 8.74 ppm. In the ^13^C NMR spectra, a doublet at 15.53 ppm (d, ^1^*J*_PC_ = 91.6 Hz) is observed. Based on the spectroscopic data, the presence of the pentavalent isomer **3** seems unlikely; however, no unambiguous assignment between the remaining possible isomeric structures **1** and **2** can be made.

**Fig. 1:**
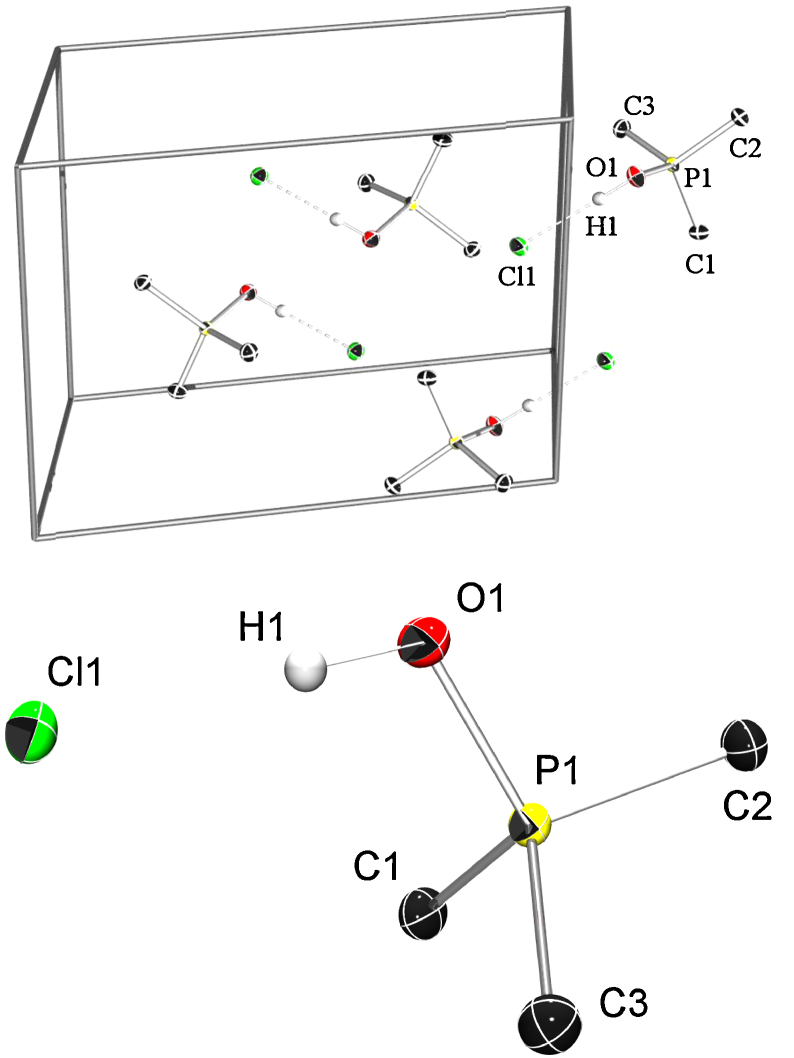
ORTEP plot of the unit cell content of **5** showing the four nearest molecules. Probability level of the thermal ellipsoids is 50%, hydrogen atoms are drawn at arbitrary radii. The hydrogen bond of the ion pairs is displayed with dotted lines. Selected distances [Å] and angles [°]: P1–O1 1.5600(4), P1–C1 1.7740(5), P1–C2 1.7725(5), P1–C3 1.7798(6), O1–H1 0.90(1), H1–Cl1 1.98(1), O1–H1···Cl1 179.5(14).

We were also able to grow single crystals suitable for X-ray diffraction from a saturated chloroform solution. The crystal structure analysis clearly shows the formation of a hydroxytrimethyl phosphonium chloride. All atoms are on general positions. All non-hydrogen atoms are refined with anisotropic displacement parameters, while the hydrogen atoms were refined isotropically without any geometric constraints. The hydrogen bond [O1–H1⋯Cl1 179.5(14)°, O1⋯Cl1 2.8763(4) Å] connects the ion pair which shows almost C_s_ symmetry. The chloride anion is surrounded by two methyl groups and the next oxygen atom is as near as 3.7421(5) Å having no significant contributions to the stabilization of the phosphonium ion pairs. Interestingly, the P–O bond is very short (P1–O1 1.5600(4) Å), which is only 4.7% longer than in Me_3_P=O (Fig. 1).

In order to account for the observed discrepancy of the calculated and the X-ray structure, we tried to involve the surroundings of the molecule in a very simple way. The very common IEF-PCM formalism for modeling a solvent by means of a polarizable continuum was used to mimic the crystallographic surroundings. For these calculations, one needs to specify a “solvent” by its dipole moment (*μ*) and the mean polarizability ($\bar{\alpha }$). Those parameters were obtained from preliminary calculations of the phosphonium ion in a highly polar medium modeled by the IEF-PCM^2^ using the Onsager equation (Eq. (4)) for polar liquids. Accounting for the different orientations of the dipole moment in the solid state gives the Kirkwood correlation factor. By using this approach, we were able model the observed structure. Interestingly also a higher correlated method – CISD – showed good agreement with the X-ray structure, but not 2^nd^ and 3^rd^ order Møller-Plesset calculations (MP2, and MP3). Table 2 gives relevant structural parameters of the calculated and the X-ray structures.

**Fig. 2:**
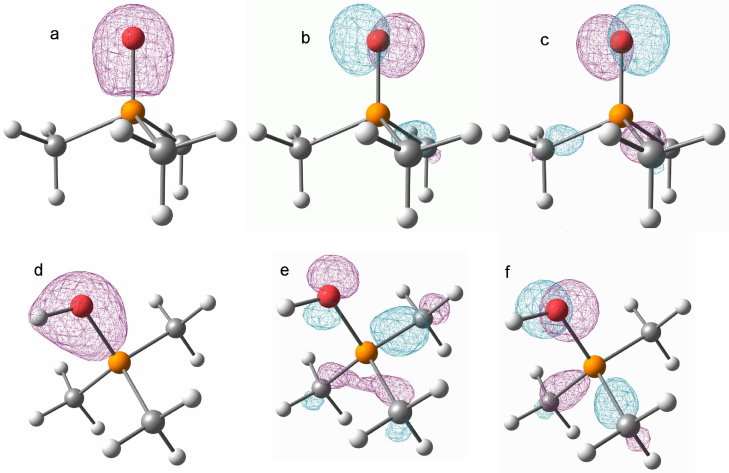
Graphical representation of selected molecular orbitals for **6** (top a–c) and **5** (bottom (d–f) at a contour level of 0.1 a.u. a: HOMO-15. b: HOMO-1. c: HOMO. d: HOMO-19. e: HOMO-4. f: HOMO-3.

A detailed analysis of the molecular orbitals of trimethyl phosphine oxide (**6**) and **5** at the B3LYP/6-311++G** level of theory with IEF-PCM (*ɛ*_R_ = 98 and *r*_solv._ = 5.32 Å) was carried out in order to understand the rather short P–O single bond. In **6** the s-type orbitals at the oxygen and phosphorus atoms form a highly polarized σ-bond (Fig. 2 a) and two p-type lone pairs of O show significant negative hyperconjugation with the P–C antibonding orbitals (Fig. 2 b + c) resulting in a short P–O bond. In compound **5**, the two σ-bonds (P–O and O–H) form two linear combinations (σ_O−H_ + σ_P−O_ (d) and σ_O−H_ – σ_P−O_), with less polarization towards the oxygen atom compared to **6**. Furthermore, the p-type orbitals of the oxygen atom show slightly diminished, but still significant negative hyperconjugation (Fig. 2 e + f). Interestingly, the interaction energy in **5** gives similar results for the solvation models (ΔG_interaction_ 3.9 kcal/mol) and the gas phase (ΔG_interaction_ 3.2 kcal/mol) despite their significantly different structures.

## 3 Conclusion

We were able to synthesize and structurally characterize the hydroxymethyl phosphonium chloride, which proves the presence of distinct ion pairs in the solid state. Different calculations referring to gas phase, solution, and “solid” state reveal a large influence of the molecular surroundings on the bonding situation besides the counter-ion which plays a crucial role for the prevailing structural isomer of these compounds. Widely implemented continuum models like the PCM were used to model the environment in the solid state for an easy prediction and confirmation of the solid state structure.

## Figures and Tables

**Table:** 

*Crystal data*	
Empirical formula	C_3_H_10_OP^+^Cl^−^
Formula weight	128.53
Crystal description	Block, colorless
Crystal size	0.28 × 0.28 × 0.18 mm
Crystal system, space group	Monoclinic, P 2_1_/*n*
Unit cell dimensions	
a	6.3264(2) Å
b	9.1397(3) Å
c	11.3681(4) Å
β	105.8420(10) °

Volume	632.35(4) Å^3^
Z	4
Calculated density	1.350 Mg/m^3^
F(000)	272
Linear absorption coefficient μ	0.735 mm^−1^
Absorption correction	Semi-empirical from equivalents
Max. and min. transmission	0.748 and 0.643
Unit cell determination	2.90 < Θ < 40.67°
	6625 reflections used at 100K

*Data collection*	
Radiation source	Fine-focus sealed tube
Radiation and wavelength	MoK_α_, 0.71073 Å
Scan type	ϕ and ω scans
Θ range for data collection	3.36 to 40.00°
Index ranges	−11 ≤ *h* ≤ 11, −15 ≤ *k* ≤ 16, −15 ≤ l ≤ 20
Reflections collected/unique	10607/3906
Significant unique reflections	3507 with *I* > 2σ(I)
R(int), R(sigma)	0.0177, 0.0194
Completeness to Θ = 40.0°	99.4%

*Refinement*	
Refinement method	Full-matrix least-squares on F^2^
Data/parameters/restraints	3906/95/0
Goodness-of-fit on F^2^	1.052
Final R indices [I > 2σ(I)]	R1 = 0.0211, wR2 = 0.0568
R indices (all data)	R1 = 0.0245, wR2 = 0.0585
Extinction expression	None
Weighting scheme	*w* = 1/[σ^2^(F_o_^2^) + (a*P*)^2^ + b*P*] where *P* = (F_o_^2^ + 2F_c_^2^)/3
Weighting scheme parameters *a*, *b*	0.0315, 0.0495
Largest Δ/σ in last cycle	0.001
Largest difference peak and hole	0.437 and −0.356e/Å^3^

**Table 1:** Structural changes on adduct formation. Comparison of selected geometric parameters of (CH_3_)_3_POHX (calculated) with (CH_3_)_3_PO (calculated and experimental). Distances in [Å] and angles in [°].

(CH_3_)_3_POHX				(CH_3_)_3_PO	
X =	F	Cl	Br	–	–
	B3LYP/6-311++G(d,p)			B3LYP/6-311++G(d,p)	X-ray [14]
P–O	1.513	1.512	1.568	1.500	1.489(6)
P–C_trans_	1.821	1.822	1.807	1.830	1.772(6)
P–C_cis_	1.823	1.823	1.808	1.830	1.772(6)
O–H	1.581	1.619	1.069	–	–
O…X	2.534	2.960	2.973	–	–
H–X	0.959	1.344	1.935	–	–
C–P–OH	10.0	34.1	180.0	–	–
POX	121.7	127.5	99.4	–	–

**Table 2:** Comparison of calculated and measured structural parameters of Me_3_POHCl.

	X-ray	B3LYP					CISD	MP2	MP3^a^
		6–311G**	6–311++G**	6–311++G**			6–311++G**	6–311++G**	6–311++G**
g.p^b^./PCM^c^		g.p.	g.p.	PCM: 0.5	PCM: 80	PCM: 98	g.p.	g.p.	g.p.
P–O	1.560(1)	1.512	1.515	1.507	1.585	1.590	1.547	1.515	1.497
P–C_trans_	1.780(1)	1.822	1.823	1.825	1.796	1.795	1.791	1.821	1.812
P–C_cis_	1.773(1)	1.823	1.821	1.826	1.803	1.802	1.791	1.823	1.811
O⋯H	0.897(14)	1.619	1.615	1.709	1.024	1.020	1.041	1.615	1.686
O⋯Cl	2.876(1)	2.960	2.580	3.020	2.916	2.931	2.787	2.958	2.989
H–Cl	1.979(14)	1.344	1.346	1.331	1.892	1.912	1.779	1.346	1.309
C–P–O–H	−176.7(9)	−145.9	−172.0	178.3	−179.7	179.9	180.0	171.9	−0.1
P–O–Cl	113.6(1)	127.5	125.9	111.5	114.4	115.3	98.2	123.4	128.5
rmsd (dist)^d^	–	**0.40**	**0.41**	**0.43**	**0.07**	**0.06**	**0.11**	**0.39**	**0.43**
rmsd (ang)^e^	–	**23.9**	**9.3**	**1.9**	**2.2**	**2.6**	**11.2**	**7.7**	**125.3**

a Calculations resulted in a staggered orientation of the C–P–O–H moiety.

b Gas phase calculations.

c Solvation model IEF-PCM with respective permittivity and *r*_solv._ = 5.32 Å.

d Root mean square deviation (rmsd) of selected distances [Å].

e rmsd of selected angles [°].

## Appendix A Computational details

All *ab initio* calculations have been carried out with the Gaussian G03 (Rev. B.04) suite of programs. All structures were fully optimized and their frequency analysis showing no imaginary frequency. The Debye-Clausius-Mosotti equation can be used for the evaluation of permittivity of molecules with a negligible permanent dipole moment. Limitations of this theory are caused by neglecting the contribution of changing the electric field induced by the permanent dipole moment:

(1) $$\frac{\varepsilon_{R}-1}{\varepsilon_{R}+2}=\frac{\rho }{M}\frac{N_{A}}{3\varepsilon_{0}}*\left(\bar{\alpha }+\frac{{\bar{\mu }}^{2}}{3*k*T}\right)$$

One of the first modifications to this theory was given by Onsager, who introduced the term “reaction field” (Eqs. (2), (3)). In brief, this is the response of the external dielectric to the dipole moment of the solute. This methodology also proved successful also for the calculation of relative permittivities of polar liquids.

(2) $$\varepsilon_{0}(\varepsilon_{R}-1)=N_{1}\frac{3\varepsilon_{R}}{2\varepsilon_{R}+1}*\frac{1}{1-\bar{\alpha }f_{R}}*\left(\bar{\alpha }+\frac{1}{1-\bar{\alpha }f_{R}}*\frac{\mu^{2}}{3kT}\right),\;\text{where}$$

(3) fR=23*N1ε0*εR−12εR+1

This approach does not account for dipole–dipole interactions. To include these interactions quantitatively, Kirkwod introduced a simple scaling factor G_K_ which reduces the molecular dipole moment to the effective dipole moment (*G*_*K*_ * *μ*_*mol*._ = *μ*_*eff*._). This factor is defined as: $G_{K}=1+\frac{1}{n}\sum_{n}\cos(\varphi_{n})$, where *n* is the number of direct neighbors and *φ*_*n*_ the angle enclosed by their respective dipole moment. For the case that the *G*_*K*_ factor becomes one, the Kirkwood equation (4) equals the Onsager equation (2).

(4) $$\varepsilon_{0}(\varepsilon_{R}-1)=N_{1}\frac{3\varepsilon_{R}}{2\varepsilon_{R}+1}*\frac{1}{1-\bar{\alpha }f_{R}}*\left(\bar{\alpha }+\frac{1}{1-\bar{\alpha }f_{R}}*\frac{G_{K}\mu^{2}}{3kT}\right)\text{,}$$

In order to determine an approximate Kirkwood factor for the crystal state, we used the dipole moment vector obtained from preliminary calculations. By alignment of the molecules (only heavy atoms) from the calculations and the X-ray structure, we obtained a translation (center of masses) and rotation matrix. The respective rotation matrices were then used to transform the computed dipole moment and calculate the angles between the different orientations in the solid state. The average of the four nearest different orientations was used to calculate the Kirkwood factor (G_K_ = 0.1412).

Solving Eq. (3) with respect to *ɛ*_*R*_ gives, in general, three solutions (one imaginary, one real negative and one positive solution, which is the physically meaningful one). The permittivity for the PCM calculations has been updated only three times showing already good convergence (*ɛ*_*R*_ = 98). The radius of the “solvent sphere” was estimated by taking the volume of the unit cell as determined from the X-ray structure divided by the number of molecules present in the cell and transforming it to a spherical shape.

## Appendix B Synthesis of **5**

All reactions were carried out under argon using modified Schlenk techniques. NMR spectra were recorded on a Varian UnityInova operating at a proton frequency of 400 MHz at 25 °C. Mass spectrometry was performed on an Agilent 5975C mass spectrometer equipped with a direct injection unit (DI-EI).

Compound **5** was synthesized according to a published procedure [8] bubbling anhydrous HCl_(g)_ through a suspension of OPMe_3_ (6.0 mmol, 0.55 g) in 100 ml Et_2_O. After filtration and removal of the solvent under reduced pressure, **5** is obtained in reasonable yields (67%, 0.52 g, 4.0 mmol). ^1^H(CDCl_3_, 400 MHz): 1.93 ppm (^3^*J*_PH_ = 18 Hz). ^13^C(CDCl_3_, 100 MHz): 15.53 ppm (*d*, ^1^*J*_PC_ = 91.6 Hz). ^31^P(CDCl_3_, 121 MHz): 66.9 ppm. EI-MS (m/z): 128.1/130.1 M^+^ (42); 92.1 Me_3_PO^+^ (85); 77.1 Me_3_PO^+^-CH_3_ (100).

## Appendix C Crystallographic details

The crystal structure of **5** was determined with a Bruker APEX-II CCD diffractometer using graphite-monochromatized Mo K_α_ radiation at 100 K. The structure was solved and refined using the SHELXL and SHELXS suite of programs [17,18]. Structure plots have been prepared using ORTEP [19]. Supplementary crystallographic data for this article can be obtained free of charge quoting CCDC 761598 from The Cambridge Crystallographic Data Centre via www.ccdc.cam.ac.uk/data_request/cif.

## Footnotes

1 The P=O bond lengths from our calculations and the X-ray structural analysis show very good agreement, though. Also the experimental P=O stretching mode (1163 cm^-1^) is in accordance with scaled/unscaled DFT results (1158/1206 cm^-1^) with a scaling factor of 0.96.

2 The continuum model IEF-PCM was used with standard parameters for water. Owing to the fact that cavities are formed as spheres over the heavy atoms only and hydrogen atoms are supposed to lie in these spheres, we used the universal force field (UFF) cavity formation algorithm.
