# Supplementary material for: Computational and experimental approaches to the molecular structure of the HCl adduct of Me3PO
Source: C R Chim. 2010 Aug;13(8-9):923–8. doi: 10.1016/j.crci.2010.07.006 (PMC3779084; doi:10.1016/j.crci.2010.07.006)
Supplement: Supplementary file 1 [file mmc1.doc]

Supplementary Material

## Computational and Experimental Approaches to the Molecular Structure of the HCl Adduct of Me3PO

Andreas Orthaber, Ferdinand Belaj and Rudolf Pietschnig*

Full list of bond lengths and angles for **5 (S2): Table S 1.**

Computational details for compounds **S1**-**S3** Me_3_POHX (**S1**: X= F, **S2**: X= Cl, **S3**: X= Br) and **S4** Me_3_P=O: Table S 2 - Table S 23.

| P(1)-O(1) | 1.5600(4) |
| --- | --- |
| P(1)-C(2) | 1.7725(5) |
| P(1)-C(1) | 1.7740(5) |
| P(1)-C(3) | 1.7798(6) |
| O(1)-H(1) | 0.897(14) |
| C(1)-H(11) | 0.993(11) |
| C(1)-H(12) | 0.956(11) |
| C(1)-H(13) | 0.921(12) |
| C(2)-H(21) | 0.973(11) |
| C(2)-H(22) | 0.951(12) |
| C(2)-H(23) | 0.925(11) |
| C(3)-H(31) | 0.952(12) |
| C(3)-H(32) | 0.948(12) |
| C(3)-H(33) | 1.001(13) |
|  |  |
| O(1)-P(1)-C(2) | 106.99(2) |
| O(1)-P(1)-C(1) | 111.47(2) |
| C(2)-P(1)-C(1) | 109.82(3) |
| O(1)-P(1)-C(3) | 110.43(3) |
| C(2)-P(1)-C(3) | 110.24(3) |
| C(1)-P(1)-C(3) | 107.90(3) |
| P(1)-O(1)-H(1) | 113.6(9) |
| P(1)-C(1)-H(11) | 107.5(6) |
| P(1)-C(1)-H(12) | 108.9(7) |
| H(11)-C(1)-H(12) | 108.0(9) |
| P(1)-C(1)-H(13) | 109.0(7) |
| H(11)-C(1)-H(13) | 113.3(9) |
| H(12)-C(1)-H(13) | 110.1(10) |
| P(1)-C(2)-H(21) | 108.0(6) |
| P(1)-C(2)-H(22) | 107.2(7) |
| H(21)-C(2)-H(22) | 114.7(9) |
| P(1)-C(2)-H(23) | 106.3(7) |
| H(21)-C(2)-H(23) | 110.0(9) |
| H(22)-C(2)-H(23) | 110.2(9) |
| P(1)-C(3)-H(31) | 109.0(7) |
| P(1)-C(3)-H(32) | 112.3(7) |
| H(31)-C(3)-H(32) | 106.9(10) |
| P(1)-C(3)-H(33) | 108.3(7) |
| H(31)-C(3)-H(33) | 110.9(10) |
| H(32)-C(3)-H(33) | 109.5(10) |

Table S 1: Selected distances [Å] and angles [°]

| No. | Atom | X | Y | Z |
| --- | --- | --- | --- | --- |
| 1 | C | -0.008289 | -0.018639 | -0.009212 |
| 2 | P | 0.017585 | 0.018542 | 1.813205 |
| 3 | O | 1.417978 | 0.003915 | 2.383526 |
| 4 | C | -0.946337 | 1.489466 | 2.291946 |
| 5 | C | -0.958643 | -1.420142 | 2.357394 |
| 6 | Cl | 3.059832 | 2.329948 | 3.193543 |
| 7 | H | -1.020753 | 1.530361 | 3.38013 |
| 8 | H | -0.425535 | 2.386848 | 1.952533 |
| 9 | H | -1.949825 | 1.466467 | 1.861143 |
| 10 | H | 0.515606 | -0.911741 | -0.35485 |
| 11 | H | -1.028915 | -0.023131 | -0.398182 |
| 12 | H | 0.519562 | 0.858231 | -0.388869 |
| 13 | H | -1.018879 | -1.415864 | 3.447185 |
| 14 | H | -1.967236 | -1.403829 | 1.938454 |
| 15 | H | -0.4506 | -2.333729 | 2.043686 |
| 16 | H | 2.357261 | 1.241502 | 2.837332 |

Table S 2: xyz coordinates for S2; B3LYP 6-311G**.

| Zero-point correction | 0.12591 |
| --- | --- |
| Thermal correction to Energy | 0.136041 |
| Thermal correction to Enthalpy | 0.136985 |
| Thermal correction to Gibbs Free Energy | 0.088461 |
| Sum of electronic and zero-point Energies | -997.167927 |
| Sum of electronic and thermal Energies | -997.157797 |
| Sum of electronic and thermal Enthalpies | -997.156853 |
| Sum of electronic and thermal Free Energies | -997.205377 |

Table S 3: Energy output for S2; B3LYP 6-311G**.

| No. | Atom | X | Y | Z |
| --- | --- | --- | --- | --- |
| 1 | C | 0.037523 | -0.04076 | 0.038248 |
| 2 | P | -0.018811 | -0.030081 | 1.860328 |
| 3 | O | 1.35458 | -0.066789 | 2.494547 |
| 4 | C | -1.059883 | -1.448115 | 2.339291 |
| 5 | C | -0.942301 | 1.465306 | 2.336995 |
| 6 | F | 3.0964 | -1.773712 | 1.804754 |
| 7 | H | -0.964328 | -0.021371 | -0.396617 |
| 8 | H | 0.561458 | -0.939732 | -0.292696 |
| 9 | H | -2.047309 | -1.401388 | 1.874265 |
| 10 | H | 0.599421 | 0.830146 | -0.304762 |
| 11 | H | -0.559232 | -2.370084 | 2.036982 |
| 12 | H | -1.932112 | 1.490736 | 1.875596 |
| 13 | H | -1.17015 | -1.456243 | 3.425407 |
| 14 | H | 2.493055 | -1.098094 | 2.120426 |
| 15 | H | -0.375216 | 2.345259 | 2.027849 |
| 16 | H | -1.047485 | 1.486116 | 3.423272 |

Table S 4: xyz coordinates for S1; B3LYP 6-311++G**.

| Zero-point correction | 0.129307 |
| --- | --- |
| Thermal correction to Energy | 0.139256 |
| Thermal correction to Enthalpy | 0.140201 |
| Thermal correction to Gibbs Free Energy | 0.092159 |
| Sum of electronic and zero-point Energies | -636.822988 |
| Sum of electronic and thermal Energies | -636.813039 |
| Sum of electronic and thermal Enthalpies | -636.812094 |
| Sum of electronic and thermal Free Energies | -636.860136 |

Table S 5: Energy output for S1; B3LYP 6-311++G**.

| No. | Atom | X | Y | Z |
| --- | --- | --- | --- | --- |
| 1 | C | 0.023961 | -0.029406 | 0.024463 |
| 2 | P | -0.013298 | -0.021924 | 1.847184 |
| 3 | O | 1.368224 | -0.050582 | 2.467509 |
| 4 | C | -0.941825 | 1.466981 | 2.33474 |
| 5 | C | -1.040326 | -1.447227 | 2.334813 |
| 6 | Cl | 3.436532 | -2.043667 | 1.761746 |
| 7 | H | -1.037553 | 1.485257 | 3.421924 |
| 8 | H | -0.383201 | 2.351078 | 2.022095 |
| 9 | H | -1.9359 | 1.486743 | 1.882192 |
| 10 | H | 0.547941 | -0.925024 | -0.315387 |
| 11 | H | -0.982831 | -0.014073 | -0.399038 |
| 12 | H | 0.57728 | 0.845113 | -0.323242 |
| 13 | H | -1.141359 | -1.45666 | 3.421799 |
| 14 | H | -2.032049 | -1.406295 | 1.878453 |
| 15 | H | -0.537419 | -2.366405 | 2.027757 |
| 16 | H | 2.53844 | -1.110579 | 2.12679 |

Table S 6: xyz coordinates for S2; B3LYP 6-311++G**.

| Zero-point correction | 0.125679 |
| --- | --- |
| Thermal correction to Energy | 0.135862 |
| Thermal correction to Enthalpy | 0.136806 |
| Thermal correction to Gibbs Free Energy | 0.087663 |
| Sum of electronic and zero-point Energies | -997.171625 |
| Sum of electronic and thermal Energies | -997.161442 |
| Sum of electronic and thermal Enthalpies | -997.160498 |
| Sum of electronic and thermal Free Energies | -997.209641 |

Table S 7: Energy output for S2; B3LYP 6-311++G**.

| No. | Atom | X | Y | Z |
| --- | --- | --- | --- | --- |
| 1 | C | -0.306314 | -1.368445 | -1.58481 |
| 2 | P | -0.315375 | -1.38184 | 0.222857 |
| 3 | C | 1.392776 | -1.369244 | 0.814451 |
| 4 | C | -1.134442 | -2.884505 | 0.802397 |
| 5 | O | -1.145702 | -0.188621 | 0.811269 |
| 6 | Br | 0.420604 | 2.082663 | -0.297184 |
| 7 | H | 0.27028 | -2.211743 | -1.971699 |
| 8 | H | 0.136986 | -0.423213 | -1.907953 |
| 9 | H | 1.948881 | -2.212535 | 0.398636 |
| 10 | H | -1.333262 | -1.42406 | -1.950577 |
| 11 | H | 1.845062 | -0.424018 | 0.503992 |
| 12 | H | -0.613074 | -3.770077 | 0.432893 |
| 13 | H | 1.396837 | -1.425337 | 1.90456 |
| 14 | H | -0.719897 | 0.744582 | 0.510038 |
| 15 | H | -2.165206 | -2.893881 | 0.444811 |
| 16 | H | -1.139362 | -2.894356 | 1.893409 |

Table S 8: xyz coordinates for S3; B3LYP 6-311++G**.

| Zero-point correction | 0.127181 |
| --- | --- |
| Thermal correction to Energy | 0.136918 |
| Thermal correction to Enthalpy | 0.137862 |
| Thermal correction to Gibbs Free Energy | 0.09144 |
| Sum of electronic and zero-point Energies | -3111.08906 |
| Sum of electronic and thermal Energies | -3111.07932 |
| Sum of electronic and thermal Enthalpies | -3111.07837 |
| Sum of electronic and thermal Free Energies | -3111.1248 |

Table S 9: Energy output for S3; B3LYP 6-311++G**.

| No. | Atom | X | Y | Z |
| --- | --- | --- | --- | --- |
| 1 | C | -0.614878 | -1.352003 | 1.127273 |
| 2 | P | -0.883158 | -0.012601 | -0.084362 |
| 3 | O | 0.065283 | -0.0679 | -1.254261 |
| 4 | C | -0.762382 | 1.540741 | 0.867734 |
| 5 | C | -2.625653 | -0.149581 | -0.610174 |
| 6 | Cl | 2.944044 | 0.06623 | -0.351885 |
| 7 | H | -1.289332 | -1.272495 | 1.982969 |
| 8 | H | 0.420646 | -1.304045 | 1.4707 |
| 9 | H | -1.4456 | 1.555554 | 1.71992 |
| 10 | H | -0.767567 | -2.313565 | 0.63336 |
| 11 | H | 0.265358 | 1.650325 | 1.220231 |
| 12 | H | -3.314682 | -0.124697 | 0.237065 |
| 13 | H | -0.988377 | 2.37939 | 0.206345 |
| 14 | H | 1.738397 | 0.002913 | -0.912023 |
| 15 | H | -2.754163 | -1.086997 | -1.154613 |
| 16 | H | -2.855296 | 0.67397 | -1.28851 |

Table S 10: xyz coordinates for S2; B3LYP 6-311++G** IEF-PCM ε_R_ = 0.5.

| Zero-point correction | | 0.125928 |  |
| --- | --- | --- | --- |
| Thermal correction to Energy | | 0.136094 |  |
| Thermal correction to Enthalpy | | 0.137038 |  |
| Thermal correction to Gibbs Free Energy | | 0.089147 |  |
| Sum of electronic and zero-point Energies | | -997.162785 |  |
| Sum of electronic and thermal Energies | | -997.152619 |  |
| Sum of electronic and thermal Enthalpies | | -997.151675 |  |
| Sum of electronic and thermal Free Energies | | -997.199566 |  |
| Variational PCM results | Kcal/mol | | |
| (Polarized solute)-solvent | 4.44 | | |

Table S 11: Energy output for S2; B3LYP 6-311++G** IEF-PCM ε_R_ = 0.5.

| No. | Atom | X | Y | Z |
| --- | --- | --- | --- | --- |
| 1 | C | -0.814622 | -1.463875 | 1.035769 |
| 2 | P | -0.941312 | -0.000088 | -0.008655 |
| 3 | O | -2.496613 | -0.009655 | -0.906969 |
| 4 | C | 0.189228 | -0.001377 | -1.119732 |
| 5 | C | -0.824684 | 1.474913 | 1.021136 |
| 6 | Cl | 2.90944 | -0.000003 | -0.069957 |
| 7 | H | -1.618483 | -1.458227 | 1.776814 |
| 8 | H | 0.150201 | -1.462055 | 1.54737 |
| 9 | H | -1.627703 | 1.470821 | 1.763095 |
| 10 | H | -0.89547 | -2.359355 | 0.415537 |
| 11 | H | 0.140554 | 1.485228 | 1.531892 |
| 12 | H | -3.329415 | -0.006282 | -0.199199 |
| 13 | H | -0.912615 | 2.363561 | 0.392107 |
| 14 | H | 1.155989 | -0.001416 | -0.78129 |
| 15 | H | -2.549307 | -0.906228 | -1.52832 |
| 16 | H | -2.552811 | 0.877989 | -1.540754 |

Table S 12: xyz coordinates for S2; B3LYP 6-311++G** IEF-PCM ε_R_ = 80.

| Zero-point correction | | 0.127714 |
| --- | --- | --- |
| Thermal correction to Energy | | 0.13769 |
| Thermal correction to Enthalpy | | 0.138634 |
| Thermal correction to Gibbs Free Energy | | 0.090258 |
| Sum of electronic and zero-point Energies | | -997.198381 |
| Sum of electronic and thermal Energies | | -997.188406 |
| Sum of electronic and thermal Enthalpies | | -997.187461 |
| Sum of electronic and thermal Free Energies | | -997.235837 |
| Variational PCM results | Kcal/mol | |
| (Polarized solute)-solvent | -30.25 | |

Table S 13: Energy output for S2; B3LYP 6-311++G** IEF-PCM ε_R_ = 80.

| No. | Atom | X | Y | Z |
| --- | --- | --- | --- | --- |
| 1 | C | 0.860229 | 1.474415 | 1.019581 |
| 2 | P | 0.958344 | -0.000366 | -0.010602 |
| 3 | O | 2.487032 | -0.009498 | -0.951262 |
| 4 | C | -0.202607 | -0.001276 | -1.096658 |
| 5 | C | 0.850211 | -1.465817 | 1.03178 |
| 6 | Cl | -2.928433 | 0.005675 | -0.018261 |
| 7 | H | 1.667508 | 1.463854 | 1.75446 |
| 8 | H | -0.099557 | 1.49024 | 1.538933 |
| 9 | H | 1.66045 | -1.457479 | 1.763387 |
| 10 | H | 0.949945 | 2.362583 | 0.392608 |
| 11 | H | -0.107624 | -1.468408 | 1.555005 |
| 12 | H | 3.337284 | -0.010707 | -0.267061 |
| 13 | H | 0.928836 | -2.35987 | 0.411757 |
| 14 | H | -1.157753 | 0.001266 | -0.738846 |
| 15 | H | 2.529117 | 0.87953 | -1.581718 |
| 16 | H | 2.52219 | -0.902914 | -1.575878 |

Table S 14: xyz coordinates for S2; B3LYP 6-311++G** IEF-PCM ε_R_ = 98.

| Zero-point correction | 0.128491 |
| --- | --- |
| Thermal correction to Energy | 0.138505 |
| Thermal correction to Enthalpy | 0.139449 |
| Thermal correction to Gibbs Free Energy | 0.091069 |
| Sum of electronic and zero-point Energies | -997.201216 |
| Sum of electronic and thermal Energies | -997.191202 |
| Sum of electronic and thermal Enthalpies | -997.190257 |
| Sum of electronic and thermal Free Energies | -997.238637 |
| Variational PCM results | Kcal/mol |
| (Polarized solute)-solvent | -34.01 |

Table S 15: Energy output for S2; B3LYP 6-311++G** IEF-PCM ε_R_ = 98.

| No. | Atom | X | Y | Z |
| --- | --- | --- | --- | --- |
| 1 | C | -0.056125 | -0.016694 | -0.039256 |
| 2 | P | -0.038817 | -0.047867 | 1.783401 |
| 3 | O | 1.359857 | -0.117351 | 2.360828 |
| 4 | C | -1.079827 | -1.462564 | 2.272261 |
| 5 | C | -0.921652 | 1.448122 | 2.33055 |
| 6 | Cl | 3.360103 | -2.13656 | 1.542511 |
| 7 | H | -1.074575 | 0.026267 | -0.431914 |
| 8 | H | 0.440363 | -0.914432 | -0.413092 |
| 9 | H | -2.08446 | -1.390892 | 1.849153 |
| 10 | H | 0.503282 | 0.854614 | -0.385258 |
| 11 | H | -0.606454 | -2.384674 | 1.929207 |
| 12 | H | -1.929108 | 1.495816 | 1.910897 |
| 13 | H | -1.146397 | -1.494327 | 3.361492 |
| 14 | H | 2.495919 | -1.192485 | 1.958234 |
| 15 | H | -0.356202 | 2.327867 | 2.017934 |
| 16 | H | -0.982212 | 1.445547 | 3.42041 |

Table S 16: xyz coordinates for S2; MP2 6-311++G**.

| Zero-point correction | 0.125656 |
| --- | --- |
| Thermal correction to Energy | 0.135839 |
| Thermal correction to Enthalpy | 0.136783 |
| Thermal correction to Gibbs Free Energy | 0.087961 |
| Sum of electronic and zero-point Energies | -997.171641 |
| Sum of electronic and thermal Energies | -997.161458 |
| Sum of electronic and thermal Enthalpies | -997.160514 |
| Sum of electronic and thermal Free Energies | -997.209336 |

Table S 17: Energy output for S2; MP2 6-311++G**.

| No. | Atom | X | Y | Z |
| --- | --- | --- | --- | --- |
| 1 | C | -2.018191 | -1.442524 | -0.32711 |
| 2 | C | -0.537133 | -0.002964 | 1.691817 |
| 3 | C | -2.015082 | 1.445562 | -0.322989 |
| 4 | H | -2.890764 | -1.419662 | 0.331523 |
| 5 | H | -1.439682 | -2.348955 | -0.132397 |
| 6 | H | -1.437529 | -0.00028 | 2.312372 |
| 7 | H | -2.348507 | -1.460845 | -1.368632 |
| 8 | H | 0.057901 | -0.891126 | 1.919001 |
| 9 | H | -2.890037 | 1.420416 | 0.332468 |
| 10 | H | 0.06465 | 0.880219 | 1.920623 |
| 11 | H | 1.876993 | -0.001281 | -0.53463 |
| 12 | H | -2.341771 | 1.469929 | -1.365557 |
| 13 | H | -1.436172 | 2.350182 | -0.121356 |
| 14 | Cl | 3.090561 | 0.000064 | -0.043755 |
| 15 | O | 0.246987 | -0.000047 | -0.965381 |
| 16 | P | -0.953872 | 0.000016 | -0.071789 |

Table S 18: xyz coordinates for S2; MP3 6-311++G**.

| Zero-point correction | 0.128419 |
| --- | --- |
| Thermal correction to Energy | 0.13854 |
| Thermal correction to Enthalpy | 0.139484 |
| Thermal correction to Gibbs Free Energy | 0.09117 |
| Sum of electronic and zero-point Energies | -995.525451 |
| Sum of electronic and thermal Energies | -995.51533 |
| Sum of electronic and thermal Enthalpies | -995.514386 |
| Sum of electronic and thermal Free Energies | -995.5627 |

Table S 19: Energy output for S2; MP3 6-311++G**.

| No. | Atom | X | Y | Z |
| --- | --- | --- | --- | --- |
| 1 | C | 0.531962 | 1.456701 | 0.976965 |
| 2 | C | 0.814049 | -0.000033 | -0.02579 |
| 3 | C | -0.058779 | 0.00005 | -1.302711 |
| 4 | H | 0.531689 | -1.456531 | 0.977233 |
| 5 | H | 2.514531 | -0.000351 | -0.587454 |
| 6 | H | -2.56052 | 0.0004 | -0.073853 |
| 7 | H | 1.181822 | 1.446409 | 1.847662 |
| 8 | H | -0.509291 | 1.462552 | 1.282818 |
| 9 | H | 1.179619 | -1.444679 | 1.84932 |
| 10 | H | 0.732093 | 2.342827 | 0.382215 |
| 11 | H | -0.510249 | -1.463504 | 1.280882 |
| 12 | H | 3.193853 | -0.001727 | 0.259883 |
| 13 | H | 0.73452 | -2.342812 | 0.383646 |
| 14 | Cl | -1.065798 | 0.000249 | -1.038774 |
| 15 | O | 2.692766 | 0.883067 | -1.19201 |
| 16 | P | 2.691681 | -0.882598 | -1.194026 |

Table S 20: xyz coordinates for S2; CISD 6-311++G**.

| Zero-point correction | 0.133818 |
| --- | --- |
| Thermal correction to Energy | 0.143194 |
| Thermal correction to Enthalpy | 0.144139 |
| Thermal correction to Gibbs Free Energy | 0.099494 |
| Sum of electronic and zero-point Energies | -995.320405 |
| Sum of electronic and thermal Energies | -995.311029 |
| Sum of electronic and thermal Enthalpies | -995.310085 |
| Sum of electronic and thermal Free Energies | -995.354729 |

Table S 21: Energy output for S2; CISD 6-311++G**.

| No. | Atom | X | Y | Z |
| --- | --- | --- | --- | --- |
| 1 | C | 0.006378 | 0.000092 | -0.008185 |
| 2 | P | -0.017386 | 0.000149 | 1.813658 |
| 3 | O | 1.374286 | 0.00025 | 2.423514 |
| 4 | C | -1.003757 | 1.453683 | 2.297707 |
| 5 | C | -1.003126 | -1.453497 | 2.298388 |
| 6 | H | -1.105729 | 1.472777 | 3.385205 |
| 7 | H | -0.488603 | 2.361383 | 1.975088 |
| 8 | H | -1.997132 | 1.422649 | 1.843343 |
| 9 | H | 0.534766 | -0.888526 | -0.361076 |
| 10 | H | -1.008797 | -0.000297 | -0.412548 |
| 11 | H | 0.534138 | 0.889023 | -0.361182 |
| 12 | H | -1.105461 | -1.471728 | 3.385876 |
| 13 | H | -1.996432 | -1.423134 | 1.8438 |
| 14 | H | -0.487601 | -2.361278 | 1.976633 |

Table S 22: xyz coordinates for S4; B3LYP 6-311++G** IEF-PCM ε_R_ = 98.

| Zero-point correction | 0.116465 |
| --- | --- |
| Thermal correction to Energy | 0.124001 |
| Thermal correction to Enthalpy | 0.124945 |
| Thermal correction to Gibbs Free Energy | 0.085881 |
| Sum of electronic and zero-point Energies | -536.346037 |
| Sum of electronic and thermal Energies | -536.338502 |
| Sum of electronic and thermal Enthalpies | -536.337558 |
| Sum of electronic and thermal Free Energies | -536.376622 |
| Variational PCM results | Kcal/mol |
| (Polarized solute)-solvent | -13.96 |

Table S 23: Energy output for S4; B3LYP 6-311++G** IEF-PCM ε_R_ = 98.
